# The usefulness of a trauma probability of survival model for forensic life-threatening danger assessments

**DOI:** 10.1007/s00414-020-02499-3

**Published:** 2021-01-03

**Authors:** Lykke Schrøder Jakobsen, Niels Lynnerup, Jacob Steinmetz, Jytte Banner

**Affiliations:** 1 Department of Forensic Medicine, Section of Forensic Pathology, University of Copenhagen, Frederik V’s Vej 11, Copenhagen East, 2100 Denmark; 2 Trauma Centre & Department of Anaesthesia, HOC, Rigshospitalet, University of Copenhagen, Blegdamsvej 9, Copenhagen East, 2100 Denmark

**Keywords:** Clinical forensic medicine, Penetrating injury, Life-threatening danger assessment, Objective injury severity, AUC-ROC

## Abstract

Clinical forensic medical examinations constitute an increasing proportion of our institution’s tasks, and, concomitantly, the authorities are now requesting forensic life-threatening danger assessments based on our examinations. The aim of this retrospective study was to assess if a probability of survival (PS) trauma score could be useful for these forensic life-threatening danger assessments and to identify a cut-off PS score as a supporting tool for the forensic practice of assessing life-threatening danger. We compared a forensic database and a trauma database and identified 161 individuals (aged 15 years or older) who had both a forensic life-threatening danger assessment and a PS score. The life-threatening danger assessments comprised the following statements: was not in life-threatening danger (NLD); could have been in life-threatening danger (CLD); or was in life-threatening danger (LD). The inclusion period was 2012–2016. A statistically significant difference was found in the PS scores between NLD, CLD and LD (chi-square test: *p* < 0.0001). The usefulness of the PS score for categorizing life-threatening danger assessments was determined by a receiver-operator characteristic (ROC) curve. The area under the curve was 0.76 (95% CI, 0.69 to 0.84) and the ROC curve revealed that a cut-off PS score of 95.8 would appropriately identify LD. Therefore, a PS score below 95.8 would indicate life-threatening danger. We propose a further exploration of how the evidence-based PS score, including a cut-off value, might be implemented in clinical forensic medical statements to add to the scientific strength of these statements.

## Introduction

Clinical forensic medical (CFM) examinations may include an assessment of the life-threatening danger of the documented injuries. This also applies to the Danish CFM examination [1], and the forensic assessments may have an impact on the police investigation and the legal aftermath of a case. The application of any protocols should ensure that board-certified forensic medical specialists follow standardized approaches; therefore, a protocol regarding the assessment of life-threatening danger was implemented in 2016 at our institution, the Department of Forensic Medicine, University of Copenhagen. Following this protocol, our forensic specialists may come to one of the following conclusions: the examined individual (1) was not in life-threatening danger (NLD) due to stable vital parameters, sparse haemorrhage, no blood transfusion, no treatment except suturing etc.; (2) could have been in life-threatening danger (CLD) because of the necessity for treatment of the injuries; or (3) was in life-threatening danger (LD) as the injuries required emergency treatment, surgery, blood transfusion etc.

The forensic life-threatening danger assessments are based on an assessment of the prior-to-treatment anatomical injuries and the subsequent health state. However, while the forensic life-threatening danger assessments are empirically grounded, they are not evidence based. Due to the nature of forensic medicine, conducting randomized clinical trials is not possible (and may not even be the proper study design) [2].

Several forensic studies have examined the applicability of trauma scoring for postmortem documentation of injuries by quantifying the injury severity at autopsy [3–8]. However, few studies have examined the potential of trauma scoring for the prediction of mortality in the CFM setting. A Swedish study with forensic participation concluded that predicting short-term mortality was possible in victims of violent assaults based on age, sex, the International Classification of Diseases Injury Severity Score (ICISS), the individual ICD 10 injury diagnoses, the anatomical location of the injuries and the cause of injury [9].

In Eastern Denmark, only CFM examinations performed at the Trauma Center at Copenhagen University Hospital (TC-CUH) are given a trauma score, with the majority having penetrating injuries (i.e. sharp force injuries and gunshot wounds). TC-CUH is one of the four trauma centres in Denmark and the only one in Eastern Denmark. Since 1999, TC-CUH has participated in the European Trauma Audit and Research Network (TARN), which was established in 1989 and is the largest European trauma database [10, 11]. In 2004, TARN presented a probability of survival (PS) model, based on data from the European trauma centres [12].

Comparison of the forensic life-threatening danger assessments and TARN-derived PS scores is interesting for two reasons. First, the PS scores are evidence based. Second, it must be a key aim in clinical forensic medicine to establish objective and rigorous methods for estimation of injury severity. Thus, our aim in the present study was to assess whether the PS scores would differ in the three forensic conclusions regarding life-threatening danger. We hypothesized that the PS score could be useful for forensic life-threatening danger assessments and that appropriate cut-off PS scores could be identified.

## Materials and methods

**Fig. 1:**
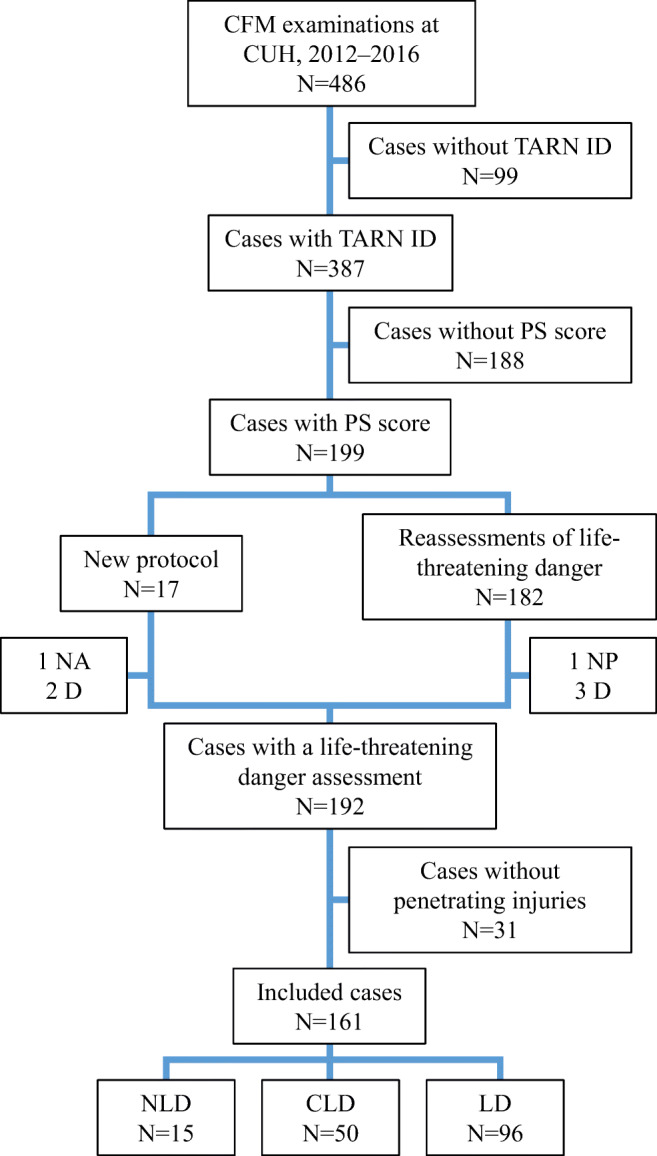
Flowchart for patient inclusion. Patients with a probability of survival (PS) score, a life-threatening danger assessment and penetrating injuries who underwent clinical forensic medical (CFM) examinations at Copenhagen University Hospital (CUH) from 2012 to 2016. Abbreviations: Trauma Audit and Research Network (TARN), cases registered in the TARN database (TARN ID), no assessment of life-threatening danger (NA), not possible to reassess the life-threatening danger (NP), died shortly after the CFM examination (D), was not in life-threatening danger (NLD), could have been in life-threatening danger (CLD), was in life-threatening danger (LD)

We identified all Eastern Danish CFM-examined individuals who were 15 years or older and the location where the CFM examination took place at Copenhagen University Hospital from January 1, 2012, to December 31, 2016. Exclusion criteria were other kinds of forensic examinations (e.g. age evaluations, torture cases and individuals examined solely for sampling of biological materials) and cases without a PS score, without a forensic life-threatening danger assessment and without penetrating injuries (Fig. 1).

We used the Danish civil registration number [13] to identify forensically examined patients registered in the TARN database at TC-CUH. In cases without a match, we manually looked up the hospital record to find a match based on age, sex, arrival date and time, and type of violence (blunt or penetrating force). A chief physician from TC-CUH controlled the matches. Not all patients included in the TARN database had a PS score due to rejection by the central TARN coder according to the TARN inclusion flow [12]. In addition to the PS score, we registered the level of consciousness according to the Glasgow Coma Scale (GCS) and the Injury Severity Score (ISS); both of these variables, together with the pre-existing medical comorbidities (PMC) [14], are used for the PS score estimation [11]. Each of the TARN variables for PS scoring carries a weighting derived from a retrospective analysis of the TARN database, which includes more than 700,000 cases and is continuously updated with data from the European trauma centres, and the variables are regularly recalibrated [12, 15].

In order to compare the PS scores with the current forensic practice regarding life-threatening danger assessments, three forensic specialists used the implemented protocol on prior-protocol cases and reassessed the life-threatening danger. The forensic specialists had the original forensic case material available: anamnesis, objective examination, obtained hospital records and police report. Thus, only the forensic report conclusion was removed. The forensic specialists stated in few cases that a reassessment was not possible most often because the hospital record had not been obtained (NP), or the examined individual had died shortly after the forensic examination but prior to the forensic report (D). These cases were excluded (cf. exclusion criteria) (Fig. 1).

The dataset was not subdivided based on the specific type of penetrating injury as a decision tree and sensitivity analysis showed no difference in the association between sharp force injuries and gunshot wounds and the life-threatening danger assessments.

### Statistical analyses

Continuous variables were reported as median values with interquartile ranges (IQRs). Categorical variables were reported as frequencies. A non-parametric Kruskal-Wallis (KW) *H* test was used [16], and in cases of a statistically significant result, a post hoc Dunn’s test was used for the pairwise comparison of the independent, categorical, life-threatening danger assessment conclusions (NLD, CLD and LD) and the dependent PS score (0–100%) [17].

The usefulness of the PS score for categorizing life-threatening danger assessments was determined by a receiver-operator characteristic (ROC) curve with an area under the curve (AUC) to evaluate the performance of the forensic protocol regarding life-threatening danger assessments of penetrating injuries [18–21]. The dichotomous outcome for the ROC analysis was NLD + CLD or LD. The most appropriate cut-off PS score was identified by determining the lower 95% fiducial limit [22, 23].

We performed all statistical analyses in SAS (SAS Enterprise Guide 7.1, 2017, SAS Institute Inc., Cary, NC, USA), and we considered a *p* value of 0.05 as statistically significant. An AUC = 0.7–0.8 was considered acceptable, an AUC = 0.8–0.9 was considered excellent, and AUC > 0.9 was considered outstanding performance [24].

## Results

We identified 486 forensically examined individuals at CUH in the 5-year study period. Of the 387 cases with a TARN submission number (TARN ID) (i.e. submitted to TARN), a central TARN coder excluded 188 of them. The remaining 199 cases had a PS score, so 161 cases were included in the final analyses as they also had an NLD, CLD or LD conclusion and documented penetrating injuries (Fig. 1).

**Fig. 2:**
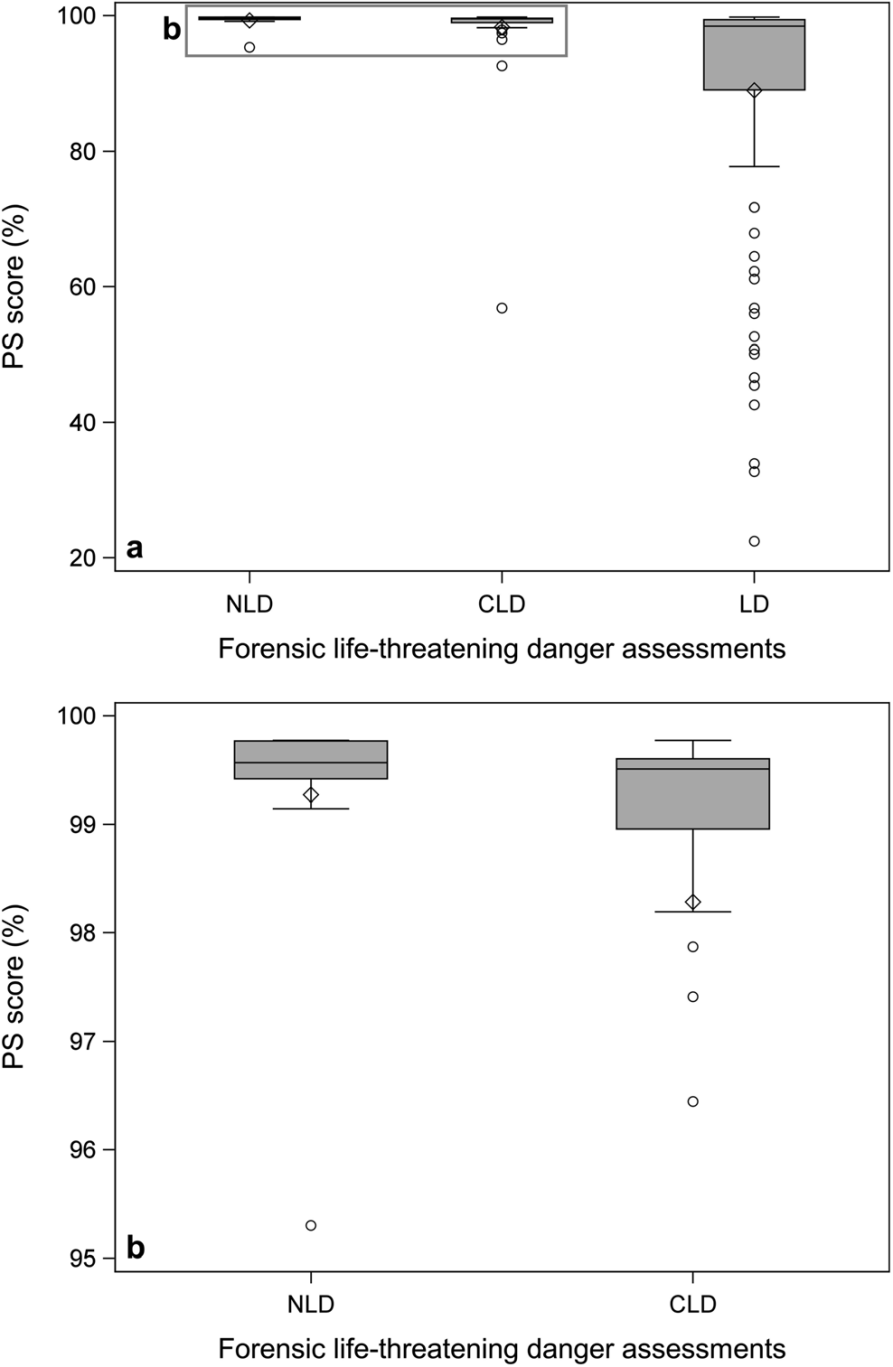
Boxplots of the forensic life-threatening danger assessments and PS scores. **a** The distribution of PS scores for NLD, CLD and LD, **b** the distribution of PS scores for NLD and CLD on upper 5% of the *y*-axis. Abbreviations: Was not in life-threatening danger (NLD), could have been in life-threatening danger (CLD), and was in life-threatening danger (LD)

In total, 14 females (median age 39, IQR 30–47 years) and 147 males (median age 28, IQR 21–38 years) were included (Table 1). The median PS score was lower for LD than for NLD and CLD (Table 1 and Fig. 2). The median PS score decreased with increasing danger severity from 99.6 to 98.4%. The LD conclusions had the lowest observed PS score and the largest range (22.4–99.8%) (Table 1).

**Table 1:** Summary statistics for the included cases

		Continuous variables								Categorical variables			
		*n*	Min	Max	Mean	SD	Median	Q1	Q3	Sex		Penetrating	
All included cases													
	Age	161	15	69	32.1	13.0	29	22	40.5	F	14	Sharp	116
	GCS	157^a^	3	15	13.2	3.7	15	14	15	M	147	Gunshot	44
	ISS	161	1	54	14.5	9.2	11	9	19			Both	1
	PS score	161	22.4	99.8	92.8	15.5	99.3	97.2	99.6				
Danger assessment conclusions													
NLD	Age	15	18	60	31.9	12.8	29	22	41	F	1	Sharp	7
	GCS	15	9	15	14.5	1.6	15	15	15	M	14	Gunshot	7
	ISS	15	1	20	7.9	4.7	9	4	10			Both	1
	PS score	15	95.3	99.8	99.2	1.1	99.6	99.2	99.8				
CLD	Age	50	15	69	30.0	12.5	26.5	21	35	F	5	Sharp	39
	GCS	49^a^	3	15	14.6	1.8	15	15	15	M	45	Gunshot	11
	ISS	50	4	34	11.7	7.1	10	5	17			Both	0
	PS score	50	56.8	99.8	98.3	6.1	99.5	99.0	99.6				
LD	Age	96	17	68	33.3	13.3	31	22.5	41	F	8	Sharp	70
	GCS	93^a^	3	15	12.2	4.4	15	11	15	M	88	Gunshot	26
	ISS	96	4	54	17.0	9.7	14	9	24.5			Both	0
	PS score	96	22.4	99.8	88.9	18.6	98.4	89.0	99.4				
Penetrating injuries													
Sharp	Age	116	15	69	33.3	13.6	30	22	41	F	14		
	GCS	115^a^	3	15	13.3	3.7	15	14	15	M	102		
	ISS	116	1	45	13.7	8.1	10	9	17.5				
	PS score	116	32.7	99.8	93.6	14.4	99.3	97.8	99.6				
Gunshot	Age	44	15	68	28.4	10	26.5	21.5	32.5	F	0		
	GCS	41^a^	3	15	12.9	3.8	15	13	15	M	44		
	ISS	44	4	54	16.9	11.3	13	9	23.5				
	PS score	44	22.4	99.8	90.6	18	99.1	90.4	99.6				

*NLD*, was not in life-threatening danger; *CLD*, could have been in life-threatening danger; *LD*, was in life-threatening danger; *PS*, probability of survival score; *F*, female; *M*, male; *sharp*, sharp force injuries; *gunshot*, gunshot wounds; *both*, both sharp force injuries and gunshot wounds; *GCS*, Glasgow Coma Scale score; *ISS*, Injury Severity Score

^a^Five individuals had no registered GSC

**Fig. 3:**
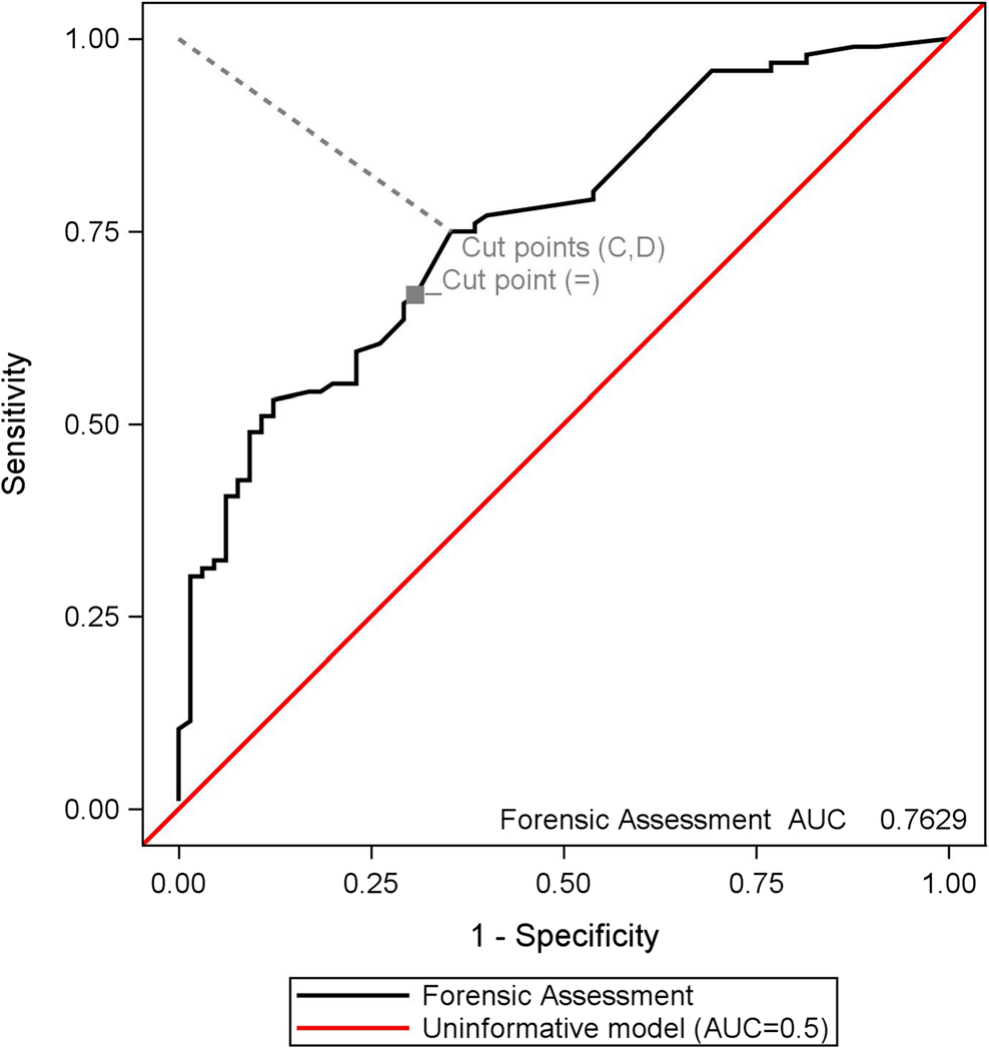
Receiver-operator characteristic curve. The probability of survival after trauma in relation to the forensic life-threatening danger assessment with cutpoints. The diagonal red line illustrates the uninformative model with an AUC = 0.5, and the dashed grey line represents the shortest distance to the upper-left corner of the graph. Abbreviations: Highest correct classification rate (*C*), minimum distance to upper-left corner (*D*), minimum absolute difference between sensitivity and specificity (=), area under the curve (AUC)

The mean ranks of PS scores showed statistically significant differences between NLD, CLD and LD, chi^2^ (2) = 33.0, *p* < 0.0001. A post hoc Dunn’s test identified LD as the reason for the statistically significant difference (Table 2). The latter supported our decision to merge NLD and CLD for the AUC-ROC analysis. The ROC curve of PS in relation to the forensic life-threatening danger assessment had an AUC at 0.76 (95% CI, 0.69 to 0.84), which was deemed acceptable [24] (Fig. 3). An appropriate cut-off PS score was identified as 95.8 (lower 95% fiducial limit).

**Table 2:** Comparison of the forensic life-threatening danger assessments and the probability of survival (PS) score

**Non-parametric analyses for PS score**								
	***n***	**Kruskal-Wallis H test**			**Post hoc Dunn’s test**			
		**Chi**^**2**^ **test**	**DF**	***p*** **value**	**Diff.**	**SE**	***q***	**Conclusion**
NLD versus CLD versus LD	161	33.02	2	< 0.0001*				
Pairwise comparison analyses								
NLD versus LD	111				52.5	12.9	4.1	Reject
NLD versus CLD	65				13.2	13.7	1.0	Do not reject
CLD versus LD	146				39.3	8.1	4.8	Reject
**ROC association statistics**								
	**Wilcoxon Mann-Whitney**				**ROC contrast test result**			
	**AUC**	**SE**	**95% CI limits**		**Chi**^**2**^ **test**		**DF**	***p*** **value**
NLD + CLD versus LD	0.76	0.04	0.69	0.84	50.38		1	< 0.0001*
**Cut-off values for PS score**								
		**Value**	**PS score**	**Cut point**	**95% fiducial limits**			
NLD + CLD versus LD								
Correct classification	C	0.71	99.30	0.51326	95.793		106.005	
Minimal distance to 0, 1	D	0.43	99.30	0.51326	95.793		106.005	
Minimal diff. (Sens − Spec)	=	0.03	99.29	0.51373	95.765		105.951	

*NLD*, was not in life-threatening danger; *CLD*, could have been in life-threatening danger; *LD*, was in life-threatening danger; Kruskal-Wallis’ and Dunn’s test *H*_0_: equal PS scores between the forensic NLD, CLD and LD conclusions. A *p* < 0.05 was considered statistically significant (*). Cutpoint *C* has the highest correct classification rate, cutpoint *D* has the minimal distance to the “perfect” point at the upper-left corner of the plot (0, 1) and cutpoint = has the minimal difference between the sensitivity and specificity

## Discussion

Based on our results, we found that the probability of survival trauma scoring could be useful for forensic life-threatening danger assessments. Furthermore, we suggest a cut-off PS score below 95.8 for use as a supporting tool for forensic determination of life-threatening danger.

The PS scores were statistically significantly lower for the LD conclusions; therefore, cases with increased mortality prediction by the PS model were forensically assessed as having been in life-threatening danger (LD). By contrast, the forensic assessments of individuals as could have been in life-threatening danger (CLD) had no statistically significant lower PS score when compared to the forensic cases assessed as having *not* been in life-threatening danger (NLD). This means that the probability of survival model cannot replace the current forensic protocol as no differentiation of the forensic NLD and CLD cases was achieved using the PS score. The PS score is the probability of survival for patients receiving the expected, proper treatment at an average trauma centre. By contrast, the forensic life-threatening danger assessments are based on prior-to-treatment anatomical injuries and subsequent health state. This distinction may explain the high PS scores, even for some of the LD cases. In addition, CLD is a hypothetical scenario and has only a forensic and legal scope of interest. Thus, it is not a relevant situation for physicians in trauma centres and may explain the lack of PS score differences between the NLD and CLD cases; from the trauma centre perspective, they are identical. It is difficult to say whether the PS scores for the CDL cases are high because of the severity of the injuries or because of a high average treatment performance; however, the admission to TC-CUH may indicate severe injuries that are treatable.

The forensic protocol performance regarding categorization of LD and NLD + CLD cases was statistically significantly better than chance (AUC 0.76, 95% CI, 0.69 to 0.84); therefore, we sought to find a cut-off PS score that could be used as a supporting tool for forensic specialists. The ideal model would have both a high sensitivity and specificity, but this is rarely the case. Therefore, the assessment of an optimal cut-off value depends on the intended use of the model; consequently, the cut-off value may vary to increase the sensitivity or specificity [21]. In our study, the assessment of an optimal cut-off PS score depended on a weighting of the importance of not missing an LD (i.e. high sensitivity) or the importance of not misclassifying an NLD + CLD case as an LD (i.e. high specificity) because of the potential legal consequences. All three cutpoint approaches had a PS score of 99.3. Choosing the lower confidence limit (fiducial limit) at 95.8 gave the most conservative cut-off PS score that would predict/identify the CFM-examined individual as having been in life-threatening danger (LD).

The inclusion of prior-protocol CFM cases necessitated a reassessment of the life-threatening danger because the criteria and conclusions changed after the protocol implementation in September 2016 [1]. Instead of comparing the previous assessment practice with the PS score, the study examined the up-to-date practice. This we consider a strength. However, due to the national legislation, forensic specialists are required to request permission to obtain a hospital record, and it is not a standardized retrieval. Thus, the information from the hospital is not always available for the forensic life-threatening danger assessments, resulting in NA or NP (Fig. 1).

Another important strength is that the snapshots regarding the CFM-examined the individual’s health state, which the forensic assessments are based on, and this can be supported by the frequently recalibrated and evidence-based PS score, which predicts the patient outcome 30 days after the trauma [15]. This is important because of on-going improvements in treatment [8, 25]. However, the continuous updates and recalibrations make the PS score time dependent, as a forensic case from 2012 might have had a different PS score if it had been evaluated after 2014 where PMC was included [14]. Thus, the forensic specialists must be aware of TARN updates and address these when using the PS score as a supporting tool.

Lastly and perhaps most importantly, we consider an important strength to be the evaluation of the performance of the forensic protocol regarding life-threatening danger assessments. At present, most of the forensic studies concerning trauma scoring have been focused on postmortem documentation and severity quantification of the injuries [3–7]. Since 2017, CFM examinations have accounted for the majority of the forensic regulatory tasks, compared to the number of autopsies. Because of this trend and the authorities’ continuously expressed request for forensic life-threatening danger assessments, the time is ripe for focusing on evidence-based validation of the forensic protocol regarding life-threatening danger assessments. Instead of identifying predictors for a multivariable model, such as in the Swedish study from 2017 [9], we have focused on the evaluations of the performance of the current forensic protocol using AUC-ROC, and we have identified a conservative cut-off PS score that can be used as a forensic supporting tool. In addition, the identified lack of a difference in PS scores between NLD and CLD raises an important question: Should the forensic protocol only surround NLD and LD conclusions and thereby refrain from the hypothetical CLD outcome?

One limitation of the present study is its use of highly selected data, which introduces selection bias. The included CFM cases with a PS score may not be representative of all CFM examinations, which are performed in many places [1]. The inclusion criteria may also explain the second limitation of this study: the relatively small number of included cases. The CFM examination may take place at a random time during the hospitalization, and because of the study inclusion flow, we missed patients transferred from other hospitals when the CFM examination was performed before this transfer (i.e. when the examination location was *not* CUH). We also only included cases with penetrating injuries as they represent the majority of the CFM-examined individuals in TC-CUH. However, even with the small number of included cases, we consider it a strength that we were able to find that the forensic protocol has an acceptable and statistically significantly better performance than an inconclusive model with AUC = 0.5.

In conclusion, we compared the forensic life-threatening danger assessments and the TARN-derived PS score and found that LD cases had statistically significantly lower mean ranks of PS scores than were obtained for the NLD and CLD cases (which showed no PS score differences). The TARN probability of survival model cannot replace the current forensic protocol, but we suggest a conservative cut-off PS score of 95.8 that can be used as a forensic supporting tool, where a PS score below this score indicates a life-threatening danger.

In perspective, the suggestion of using a cut-off PS score in the CFM setting requires a scientific evaluation of its value as a supporting tool. A future prospective study should examine how the PS score might influence forensic specialists’ assessments of life-threatening danger and potentially decrease those specialists’ uncertainty regarding these assessments. Another reasonable investigation might be to examine the impact of forensic life-threatening danger assessments on the legal aftermath.

## Data Availability

Not applicable.

## References

[1] Jakobsen LS, Jacobsen C, Lynnerup N, Steinmetz J, Banner J (2020) Clinical forensic medicine in Eastern Denmark: organisation and assessments. Med Sci Law:25802419898338. 10.1177/0025802419898338 10.1177/0025802419898338 32090675

[2] Sackett DL, Rosenberg WM, Gray JA, Haynes RB, Richardson WS (1996). Evidence based medicine: what it is and what it isn’t. BMJ.

[3] Friedman Z, Kugel C, Hiss J, Marganit B, Stein M, Shapira SC (1996). The Abbreviated Injury Scale. A valuable tool for forensic documentation of trauma. Am J Forensic Med Pathol.

[4] Sharma BR (2005). The Injury Scale – a valuable tool for forensic documentation of trauma. J Clin Forensic Med.

[5] Leth PM, Ibsen M (2010). Abbreviated Injury Scale scoring in traffic fatalities: comparison of computerized tomography and autopsy. J Trauma.

[6] Tamsen F, Sturup J, Thiblin I (2017). Quantifying homicide injuries: a Swedish time trend study using the Homicide Injury Scale. Scand J Forensic Sci.

[7] Boudreau RM, O’Neal E, Besl KM, Gordon SJ, Ralston W, Elterman JB, Pritts TA, Robinson BRH (2019). do autopsies still matter? The influence of autopsy data on final injury severity score calculations. J Surg Res.

[8] Thomsen AH, Villesen P, Brink O, Leth PM, Hougen HP (2020). Improved medical treatment could explain a decrease in homicides with a single stab wound. Forensic Sci Med Pathol.

[9] Gedeborg R, Svennblad B, Byberg L, Michaelsson K, Thiblin I (2017). Prediction of mortality risk in victims of violent crimes. Forensic Sci Int.

[10] Web group at Rigshospitalet (n.d.) Traumecenter og Akut modtagelse - Dansk Traume Register og Trauma Audit and Research Network. https://www.rigshospitalet.dk/afdelinger-og-klinikker/hovedorto/traumecenter-og-akut-modtagelse/for-fagfolk/Sider/procedure-ved-overflytning.aspx. Accessed 24 March 2020

[11] Lecky F, Woodford M, Edwards A, Bouamra O, Coats T (2014). Trauma scoring systems and databases. Br J Anaesth.

[12] Trauma Audit and Research Network (2019) The TARN Probability of Survival Model. https://www.tarn.ac.uk/Content.aspx?ca=4&c=3515. Accessed 24 March 2020

[13] Bekendtgørelse af lov om Det Centrale Personregister [Ministerial order of the Civil Registration System Act ] (2020) vol LBK nr 1297 af 03/09/2020, j.nr. 2020-8466. Ministry of Social Affairs and the Interior, Copenhagen

[14] Charlson ME, Pompei P, Ales KL, MacKenzie CR (1987). A new method of classifying prognostic comorbidity in longitudinal studies: development and validation. J Chronic Dis.

[15] Bouamra O, Jacques R, Edwards A, Yates DW, Lawrence T, Jenks T, Woodford M, Lecky F (2015). Prediction modelling for trauma using comorbidity and ‘true’ 30-day outcome. Emerg Med J.

[16] Lund Research Ltd (2018) Statistics Laerd, Kruskal-Wallis H Test, Background & Requirements. https://statistics.laerd.com/. Accessed 24 September 2020

[17] Elliott AC, Hynan LS (2011). A SAS((R)) macro implementation of a multiple comparison post hoc test for a Kruskal-Wallis analysis. Comput Methods Prog Biomed.

[18] Hanley JA, McNeil BJ (1982). The meaning and use of the area under a receiver operating characteristic (ROC) curve. Radiology.

[19] Zhou X, Obuchowski NA, McClish DK, Zhou X, Obuchowski NA, McClish DK (2002). Measures of diagnostic accuracy: The ROC curve. Statistical methods in diagnostic medicine.

[20] Zhou X, Obuchowski NA, McClish DK, Zhou X, Obuchowski NA, McClish DK (2002). Measures of diagnostic accuracy: the area ander the ROC curve. Statistical methods in diagnostic medicine.

[21] Akobeng AK (2007). Understanding diagnostic tests 3: Receiver operating characteristic curves. Acta Paediatr.

[22] SAS Institute Inc (2020) SAS Usage Note 45339: plot and compare ROC curves from logistic models fit to independent samples. https://support.sas.com/kb/45/339.html. Accessed 22 September 2020

[23] SAS Institute Inc (2020) SAS sample 25018 Plot ROC curve with cutpoint labeling and optimal cutpoint analysis. https://support.sas.com/kb/25/018.html. Accessed 22 September 2020

[24] Mandrekar JN (2010). Receiver operating characteristic curve in diagnostic test assessment. J Thorac Oncol.

[25] Larsen R, Bäckström D, Fredrikson M, Steinvall I, Gedeborg R, Sjoberg F (2018). Decreased risk adjusted 30-day mortality for hospital admitted injuries: a multi-centre longitudinal study. Scand J Trauma Resusc Emerg Med.

